# Invasion of Africa by a single *pfcrt *allele of South East Asian type

**DOI:** 10.1186/1475-2875-5-34

**Published:** 2006-04-26

**Authors:** Frédéric Ariey, Thierry Fandeur, Remy Durand, Milijaona Randrianarivelojosia, Ronan Jambou, Eric Legrand, Marie Thérèse Ekala, Christiane Bouchier, Sandrine Cojean, Jean Bernard Duchemin, Vincent Robert, Jacques Le Bras, Odile Mercereau-Puijalon

**Affiliations:** 1 Institut Pasteur du Cambodge, Phnom-Penh, Cambodia; 2 UMR Université-INRA d'Immunologie Parasitaire, Tours, France; 3 Université Paris13, EA 3406, Paris, France; 4 Institut Pasteur de Madagascar, Antananarivo, Madagascar; 5 Institut Pasteur de Dakar, Sénégal; 6 Institut Pasteur de Guyane Française, Cayenne, France; 7 Institut Pasteur de Paris, France; 8 CERMES, Niamey, Niger; 9 Institut de Recherche pour le Développement and Muséum National d'Histoire Naturelle, Paris, France; 10 CNRCP, APHP & Université Paris 5, EA 209, Paris, France

## Abstract

**Background:**

Because of its dramatic public health impact, *Plasmodium falciparum *resistance to chloroquine (CQ) has been documented early on. Chloroquine-resistance (CQR) emerged in the late 1950's independently in South East Asia and South America and progressively spread over all malaria areas. CQR was reported in East Africa in the 1970's, and has since invaded the African continent. Many questions remain about the actual selection and spreading process of CQR parasites, and about the evolution of the ancestral mutant gene(s) during spreading.

**Methods:**

Eleven clinical isolates of *P. falciparum *from Cambodia and 238 from Africa (Senegal, Ivory Coast, Bukina Faso, Mali, Guinea, Togo, Benin, Niger, Congo, Madagascar, Comoros Islands, Tanzania, Kenya, Mozambique, Cameroun, Gabon) were collected during active case detection surveys carried out between 1996 and 2001. Parasite DNA was extracted from frozen blood aliquots and amplification of the gene *pfcrt *exon 2 (codon 72–76), exon 4 and intron 4 (codon 220 and microsatellite marker) were performed. All fragments were sequenced.

**Results:**

124 isolates with a sensitive (c76/c220:CVMNK/A) haplotype and 125 isolates with a resistant c76/c220:CVIET/S haplotype were found. The microsatellite showed 17 different types in the isolates carrying the c76/c220:CVMNK/A haplotype while all 125 isolates with a CVIET/S haplotype but two had a single microsatellite type, namely (TAAA)3(TA)15, whatever the location or time of collection.

**Conclusion:**

Those results are consistent with the migration of a single ancestral *pfcrt *CQR allele from Asia to Africa. This is related to the importance of PFCRT in the fitness of *P. falciparum *point out this protein as a potential target for developments of new antimalarial drugs.

## Background

The molecular basis of chloroquine resistance has recently been elucidated. *Pfcrt *(*Plasmodium falciparum *chloroquine resistance transporter), a single copy, 13-exon gene, localized on chromosome 7, codes for a parasite digestive vacuole trans-membrane protein, which plays a key role in CQR [1,2]. The *pfcrt *gene appears to be polymorphic and more than 20 different amino-acid sequences have been identified [3-5]. Recent genome wide microsatellite scanning has shown marked linkage disequilibrium around the *pfcrt *locus and pointed to four distinct founder events, with one ancestral mutant originating in Asia and subsequently invading Africa [6]. The evolution of this mutant after its local emergence and its spread across Africa has not been studied.

**Figure 1:**
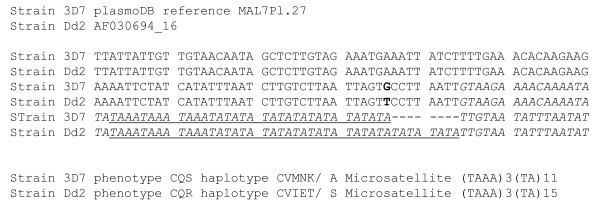
***pfcrt *genomic sequence available in EMBL**. In bold, the mutation leading to the amino acid change in 220 position, in italic the beginning of the 4th intron of the *pfcrt *gene showing the (TAAA)m(TA)n microsatellite (underscore).

Specific sets of mutations in *pfcrt *have been associated with chloroquine resistance. All but one *in vitro *CQ-sensitive (CQS) isolates carry a CVMNK haplotype at residues 72–76, whereas *in vitro *CQR isolates and isolates collected from CQ treatment failures harbour a different 72–76 haplotype. Strikingly, in spite of numerous studies done in Africa, only one resistant haplotype (CVIET) has been ever found so far in this continent. This haplotype carries in addition to the set of mutations in codons 72–76 within exon 2, a A to S mutation at codon 220 located within exon 4, resulting in a CVIET/S haplotype [7]. Nine bp downstream from this mutation starts intron 4, which contains a polymorphic (TAAA)n(TA)m microsatellite (Figure 1). Because microsatellites evolve much faster than coding sequences, intron-associated microsatellite polymorphism can be used to ear-mark individual *pfcrt *alleles and to pin-point the spread of specific alleles. In order to test the 'one event invasion theory', a characterization of CVMNK/A and CVIET/S alleles from Asia and Africa was carried out.

## Methods

### Sampling

Eleven clinical isolates of *P. falciparum *from Cambodia, 112 from West and Central Africa (Senegal, Ivory Coast, Bukina Faso, Mali, Guinea, Togo, Benin, Niger, Congo) and 126 from East and South Africa (Madagascar, Comoros Islands, Tanzania, Kenya, Mozambique, Cameroun, Gabon) were collected during active case detection surveys carried out between 1996 and 2001. Briefly, 5 mL blood samples were collected in EDTA vacutainers from patients diagnosed as having uncomplicated *P. falciparum *malaria. Immediately after blood collection, the patients were treated according to national recommendations. Blood samples were kept at 4°C for *in vitro *drug sensitivity testing. An aliquot of each isolate was frozen at -20°C for molecular analysis. Informed consent was obtained from the patients. Ethical clearance was obtained from the respective National Ethical Commitees.

### DNA extraction, PCR analysis and sequencing

Parasite DNA was extracted from frozen blood aliquots by the phenol-chloroform method as described previously [8]. The *pfcrt *haplotype was determined by sequencing PCR fragments, amplified from single clone infections (as determined using *msp2 *amplification profiles see [8] for detail) using two sets of primers designed to amplify a 194 bp exon 2 fragment (CRT76-sense 5'-GGTGGAGGTTCTTGTCTTGG-3 and CRT76-antisense 5'-ATAAAGTTGTGAGTTTCGGATG-3') and 309 bp fragment from exon 4 to exon 5 (CRT220-sense 5'-TTATACAATTATCTCGGAGCAG-3' and CRT220-antisense 5'-CATGTTTGAAAAGCATACAGGC-3'). The primers were selected on the basis of the sequence of the *pfcrt *gene of the Dd2 clone (accession number AF030694). PCR was performed in a PTC-100 thermal cycler (MJ Research, Merck Eurolab) under the following conditions: 50 μL final volume containing 2 μL DNA, 200 μM each dNTP, 0.5 μM each primer, 2 mM MgCl2, and 2 U *Taq*I DNA polymerase (Promega) in the buffer supplied by the manufacturer.

The amplification conditions were: 1 min at 95°C, 1 min 30 at 59°C, and 1 min at 72°C for 1 cycle; 10 s at 95°C, 1 min 30 at 59°C, and 2 min at 72°C for 35 cycles, and a final extension step at 72°C for 10 min. The resulting PCR products contained codons 76 and 220 as well as intron 4, respectively. All fragments were subjected to gel electrophoresis on agarose gels containing ethidium bromide and were sequenced using the fluorescent dye terminator method as described in [9]. The multiple infections still found on the basis of sequencing of the microsatelittes were discarded from the analysis (two Cambodian and six African isolates). Each sequence was analysed using Chromas^® ^v 1.45 software.

## Results

**Figure 2:**
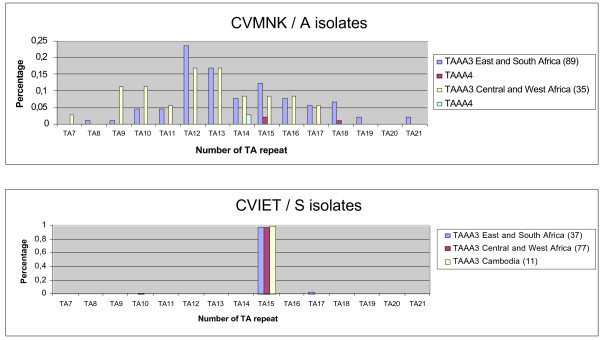
***pfcrt *intron 4 microsatellite allelic frequencies in different populations**. The relative frequency of the various allelic forms within each haplotype subgroup is indicated. Number of CVMNK/A isolates investigated was 89 from East and Austral Africa and 35 from West and Central Africa. Number of CVIET/S isolates investigated was 37 from East and Austral Africa, 77 from West and Central Africa and 11 from Cambodia. (TAAA)m(TA)n alleles are classified according to the number (n) of TA repeats that follow (TAAA)3 (plain bars) or (TAAA)4 (hatched bars). The colour codes for the various geographical regions are blue for East and Austral Africa, red for West and Central Africa and yellow for Cambodia.

Briefly, 124 isolates with a sensitive (CVMNK/A) haplotype and 125 isolates with a resistant CVIET/S haplotype were typed for the intron 4 microsatellite. Polymorphism of this locus was large, with total of 17 distinct microsatellite alleles identified, namely forteen (TAAA)3 (TA)7-21 and three (TAAA)4 (TA)11-18 (Figure 2). All 17 types were detected in the isolates carrying the CVMNK/A haplotype. Thus, numerous distinct alleles carry the "wild type" CQS haplotype. The individual distribution of the various microsatellite types was consistent with a random generation of polymorphism, as expected for microsatellites and there were no indication of clustering. In contrast, all 125 isolates with a CVIET/S haplotype but two had a single microsatellite type, namely (TAAA)3(TA)15, whatever the location or time of collection. The same microsatellite type was found in the CQR Dd2 clone of the IndochinaIII strain used for the original characterization of the resistant CVIET/S allele (Figure 1). On the other hand, the (TAAA)3(TA)11 type of the CQS 3D7 reference clone was observed in only six of 124 field isolates with the CVMNK/A haplotype, consistent with the extensive *Pfcrt *intron 4 polymorphism in CQS isolates.

The strikingly different microsatellite distribution in isolates carrying a resistant or a sensitive haplotype was highly significant (chi-square test *P *< 0.00001). Intron 4 microsatellite field polymorphism is such that the observation of a single allelic form in all but two isolates carrying a resistant haplotype cannot be explained by chance. This indicates that the resistant CVIET/S haplotype is indeed ear-marked by a specific intragenic microsatellite, and points to the existence of a single progenitor allelic form for this locus in African areas investigated here. This represents a strong population genetic evidence for an absence of endogenous emergence of a resistant allele in Africa. It demonstrates the migration of one single CVIET/S *pfcrt *allele from South East Asia into Africa and its subsequent spreading throughout the whole African continent.

## Discussion

Interestingly, a similar analysis on 15 CQR isolates from French Guiana showed that the typical South American (SVMNT/S) resistant haplotype was also associated with a single (TAAA)3(TA)14 microsatellite type, that differed from the (TAAA)3(TA)15 observed in African and Asian CQR isolates. This indicates that the microsatellite sequence itself has limited phenotypic impact and is reminiscent with the association of resistant *pfcrt *haplotypes with specific intron 4 microsatellites. This shows that resistant haplotypes that have emerged independently in Asia and in South America more than 30 years ago have limited local diversity nowadays.

### Why did resistant parasites emerge in Asia/America and not in Africa, despite the larger size of the overall African *P. falciparum *population?

One of the explanations could be that there is higher *P. falciparum *mutation rate in Asia/America than in Africa [10]. But, if so, after two decades without CQ pressure in Asia/America, *falciparum pfcrt *would have "reversed" to the wild type allele, since the appearance of resistant strains occurred at the very beginning of the introduction of chloroquine on a large scale. The latest studies, that were performed in Asia/America, show no reappearance of wild type *pfcrt *strains, invalidate this hypothesis [4].

Most likely, this emergence can be attributed to differences in transmission conditions and population structure. The endemic areas in South East Asia and South America can be considered as "localized permanent epidemics" [11], with a metapopulation structure for the *P. falciparum *populations, in which forces acting on differentiation are more effective than in Africa [12]. Indeed population isolation is thought to lead to an increased parasite diversity or local sub-speciation. In a subpopulation of a small number of parasites, genetic drift will act as a major force. This will increase polymorphism on coding sequence without any link with the possible fitness of the selected allele (this also as been shown for *cytochrome b *sequence in Asia/America compared to Africa [13]). When CQ pressure increased, some *pfcrt *alleles associated with low fitness in CQ free environment will become associated with a high fitness in this CQ environment and could thus invade the African parasite population. If CQ pressure stops, and if the ''wild type allele" is still present in the subpopulation, it will replace the mutant allele (studies in Malawi show that the relative fitness of the mutant *pfcrt *allele in the absence of CQ pressure is 0.76 or 0.86 [15,16]).

## Conclusion

Field isolates collected from clinical cases confirm that the current continent-wide CQR in Africa is due to the invasion of a single mutant allele. Therefore, monitoring the gene flow regionally can provide valuable information in assessing selection of candidate resistance gene by selective drug pressure for other antimalarials or vaccine escape.

Moreover, despite the overall PfCRT protein polymorphisms described so far in Asia [4], only one major allele has invaded Africa. This can probably be explain by the importance of this protein in the fitness of the parasite and thus to the difficulty for a *pfcrt *mutant to be selected in CQ-free area of high transmission where fierce competition occurs between strains. This strongly suggest that *pfcrt *is still one of the major potential target for antimalarial drugs and reinforces the need for a better understanding of its role in *Plasmodium *metabolism [17].

## Competing interests

The author(s) declare that they have no competing interests.

## Authors' contributions

FA, TF, RD, MR, RJ, EL, JBD, VR, JLB and OMP paritcipate to the sampling, the molecular typing of isolates, the data analysis and contributed for the elaboration of manuscript. MTE, CB and SC performed the molecular analysis of the majority of the samples. All authors read and approved the final manuscript.
